# Abstracts from the Annual Conference on Aging, Mayan Ranch, Texas Hill Country, Bandera, Texas, USA, 2013

**DOI:** 10.3402/pba.v3i0.23389

**Published:** 2013-12-11

The San Antonio Nathan Shock Center Conferences have attracted international speakers and participants since 1995. This annual conference, held in Bandera, Texas, addresses a different topic in the biology of aging each year. The venue's intimate setting, relatively remote location and common areas are ideal for a small conference (80–100 participants) where informal intellectual interchange supplements that of the formal sessions. The 2013 meeting, part of an annual series sponsored by the Nathan Shock Center of Excellence in the Biology of Aging and the Barshop Institute for Longevity and Aging Studies at the University of Texas Health Science Center San Antonio, addressed the concept that stem cells play a role in healthy aging.

Abstracts from posters presented at the meeting are presented in this special proceedings issue to provide an overview of the breadth and depth of the program. The abstracts are organized into four session topics, which include: (1) The neurogenic niche; (2) Muscle stem cells in aging; (3) Reprogramming in aging; and (4) Better aging through stem cells.

*Peter Hornsby, PhD*

*Erzsebet Kokovay, MD, PhD*

*Vivienne Rebel, PhD*

*Holy Van Remmen, PhD*

*Christi Walter, PhD*

Conference organizers

