# Supplementary material for: Abstracts from the Annual Conference on Aging, Mayan Ranch, Texas Hill Country, Bandera, Texas, USA, 2013
Source: Pathobiol Aging Age Relat Dis. 2013 Dec 11;3:10.3402/pba.v3i0.23389. doi: 10.3402/pba.v3i0.23389 (PMC3864161; doi:10.3402/pba.v3i0.23389)
Supplement: Related texts

# Sub article: Butyrate prevents skeletal muscle atrophy during aging

**Published:** 2013-12-11

**Authors:** Michael E. Walsh, Arunabh Bhattachrya, Yuhong Liu, Lauren Sloan, Holly Van Remmen

**Affiliations:** 1 Department of Cellular and Structural Biology, University of Texas Health Science Center at San Antonio, San Antonio, TX, USA; 2 Barshop Institute for Longevity and Aging Studies, University of Texas Health Science Center at San Antonio, San Antonio, TX, USA; 3 South Texas Veterans Health Care System, San Antonio, TX, USA

## Abstract

Motor neurons form a specialized synapse with skeletal muscle known as the neuromuscular junction (NMJ), and degeneration of the NMJ has been implicated in disease and aging. Histone deacetylases mediate NMJ-regulated gene transcription and are involved in neurogenic muscle atrophy, although their role in age-related muscle atrophy is not known. HDAC4 and HDAC5 knockout mice are protected against surgical denervation, and pharmacological inhibition of histone deacetylases is protective in multiple models of neuromuscular disease. In this study, we examined the effect of butyrate, a histone deacetylase inhibitor, on muscle atrophy during sciatic nerve crush and age-related muscle atrophy. We demonstrate that butyrate increases histone acetylation *in vivo* and protects against the muscle loss induced by sciatic nerve crush and aging. Control-fed mice lost 22% of their gastrocnemius mass while the butyrate-fed mice lost only 11% one week after sciatic nerve crush surgery. Butyrate protects against the loss of cross-sectional area, increases catalase and MnSOD activity, and reduces oxidative damage during nerve crush. Consistently, butyrate protects against age-related muscle atrophy in mice by modulating antioxidant activity, reducing oxidative damage, and increasing mitochondrial biogenesis. We also report improved metabolism in old mice fed butyrate, including improved glucose tolerance and increased whole-body oxygen consumption. Future studies will determine the mechanism by which butyrate protects against age-related muscle atrophy.

# Sub article: Rapamycin attenuates motor deficits in neuronal A53T human α-synuclein transgenic mice and reduces 4-hydroxynonenal modified proteins

**Published:** 2013-12-11

**Authors:** Xiang Bai, Margaret Chia-Ying Wey, Elizabeth Fernandez, Randy Strong

**Affiliations:** 1 Department of Pharmacology, University of Texas Health Science Center, San Antonio, TX, USA; 2 Barshop Institute for Longevity and Aging Studies, University of Texas Health Science Center, San Antonio, TX, USA; 3 Center for Biomedical Neurosciences, University of Texas Health Science Center, San Antonio, TX, USA; 4 Geriatric Research, Education and Clinical Center, South Texas Veterans Health Care Network, San Antonio, TX, USA

## Abstract

Synucleinopathies, including Parkinson's disease (PD), multiple system atrophy and dementia with Lewy bodies, are age-related neurodegenerative disorders characterized by pathological a-synuclein inclusions. Synucleinopathies have common motor deficits and affect millions of patients worldwide. The A53T human a-synuclein mutation is linked to both sporadic and familial PD. Rapamycin, an mTOR inhibitor, reduces human a-synuclein accumulation and neurodegenerative phenotype *in vitro* and *in vivo*. Long-term feeding of a rapamycin diet extends mouse lifespan and the mechanisms are hypothesized to be mediated via delaying age-related diseases including PD. The aim of the study is to determine whether long-term feeding rapamycin diet at the dose that extends mouse lifespan attenuates motor deficits in neuronal A53T a-synuclein transgenic mice. A diet containing microencapsulated rapamycin (14 ppm in diet; 2.25 mg/kg body weight/day) or the microencapsulation polymer was fed to age-matched wild-type and A53T mice from 13 weeks of age. After 24 weeks of treatment, rapamycin improved performance on forepaw stepping adjustment test, accelerating rotarod test and pole test in both genders. Rapamycin also increased front stride length in male A53T mice. Total human a-synuclein levels in midbrain, striatum, brain stem, cerebellum, and spinal cord were not altered by rapamycin. Oxidative stress plays an important role in pathogenesis of neurodegenerative diseases. The lipid peroxidation product 4-hydroxynonenal has been detected in the postmortem brain of PD patients and is believed to contribute to the neurotoxic effects of a-synuclein. Levels of 4-hydroxynonenal protein adducts were significantly decreased in midbrain, striatum, and spinal cord of both genders of A53T mice as well as brain stem and cerebellum of female A53T mice. In conclusion, long-term rapamycin treatment attenuated motor deficits and reduced 4-hydroxynonenal in the central nervous system regions that subserve motor function and motor coordination in the A53T mice.

# Sub article: The protective role of calcineurin CN-A beta in ER stress via activation of the unfolded protein responses in astrocytes

**Published:** 2013-12-11

**Authors:** Yanan Chen, Mariana Bollo, Deborah Holstein, James D. Lechleiter

**Affiliations:** 1 Department of Cellular and Structural Biology, University of Texas Health Science Center at San Antonio, San Antonio, TX, USA; 2 Instituto de Investigación Médica Mercedes y Martín Ferreyra (INIMEC CONICET), Universidad Nacional de Córdoba, Argentina

## Abstract

The accumulation of unfolded proteins in the endoplasmic reticulum (ER) activates a signal transduction cascade called the unfolded protein response (UPR). The most immediate response is the attenuation of protein synthesis, initiated by autophosphorylation of PKR-like ER kinase (PERK). Recently, our group reported that calcineurin (CN) strengthened the UPR by binding to PERK and enhancing its autophosphorylation (Bollo et al. PLoSOne 5 (8): e11925). Here, we report that CN-A beta protects astrocytes from ER stress, likely by enhancing autophosphorylation of PERK. First, we found that levels of phosphorylated-PERK and CN-A beta were significantly increased in astrocytes within 1 hour of oxygen and glucose deprivation (OGD). Second, overexpression of CN-A beta significantly increased the viability of wild-type astrocytes during OGD (1 hr), but not that of PERK^−/ −^ astrocytes. Third, co-immunoprecipitation showed that CN-A beta preferentially interacted with PERK in ER-stressed astrocytes. Fourth, experiments with recombinant proteins demonstrated that PERK autophosphorylation and oligomerization were increased in the presence of CN-A beta. Finally, rapamycin-induced dimerization of CFP-FRB-cytochrome5 (ER anchor) and YFP-FKBP-CN-A beta inside human astrocytes increased PERK phosphorylation. Taken together, we suggest a novel physiological function of the classic phosphatase CN-A beta is to bind PERK and enhance the early UPR.

# Sub article: Inflammation in the aging neural stem cell niche

**Published:** 2013-12-11

**Authors:** Rene Solano Fonseca, Rekha Raghunathan, Erzsebet Kokovay

**Affiliations:** Department of Cellular and Structural Biology, University of Texas Health Science Center at San Antonio, San Antonio, TX, USA

## Abstract

The subventricular zone (SVZ) is the largest reservoir of neural stem cells (NSCs) in the adult brain. Neurogenesis is reduced during aging and this decline in NSC function is thought to contribute to reduced brain function. Here, we propose immune activation and cellular senescence as contributors to age-related declines in NSC function. We observed a sharp decrease in neurogenesis in mice between the ages of 6 and 12 months. Neuroblast number was also reduced with age. We observed the appearance of β-galactosidase-positive cells, a marker of cellular senescence concomitant with reductions in neurogenesis. Senescent cells secrete factors into the extracellular environment, a phenomenon known as the senescence associated secretory phenotype (SASP). The SASP has been associated with upregulated inflammation and immune activation due to proinflammatory factors. Microglia are the prominent immune cells in the brain. Microglial activation results in the release of proinflammatory cytokines. There was no change in the number or percent of microglia cells relative to other cells in the SVZ during aging. However, both morphological changes and increased CD68 expression were observed during aging indicative of microglia becoming more activated. Interestingly, a short-term diet of encapsulated rapamycin, an inhibitor of the mTOR pathway, resulted in reduced cellular senescence in the aged brain compared to the young and an overall increase in NSC proliferation. Our results suggest that during aging there is increased inflammation in the SVZ, which may be an important contributor to declines in neural stem cell function.

# Sub article: Increased NLRP3 inflammasome expression correlates with cognitive decline in animals exposed to a mitochondrial ROS inducer

**Published:** 2013-12-11

**Authors:** Sealy Hambright, Yan Lai, Show-Li Chen, Qitao Ran

**Affiliations:** 1 Department of Cellular and Structural Biology, University of Texas Health Science Center at San Antonio, TX, USA; 2 South Texas Veterans Health Care System, San Antonio, TX, USA; 3 Barshop Institute for Longevity and Aging Studies, University of Texas Health Science Center at San Antonio, San Antonio, TX, USA

## Abstract

Increased mitochondrial reactive oxygen species (ROS) is believed to play a key role in cognitive decline associated with aging and Alzheimer's disease, but the mechanism remains unclear. Inflammasomes are multiprotein oligomers that recognize damage-associated molecular patterns (DAMPs) to activate caspase-1, thereby allowing for cleavage and subsequent release of proinflammatory cytokines IL-1β and IL-18. Among the inflammasomes, the NLRP3 inflammasome plays a key role in sensing oxidative stress such as increased mitochondrial ROS. We are interested in studying the role of mitochondrial ROS in cognitive decline associated with aging and Alzheimer's disease using mouse models exposed to paraquat, a mitochondrial ROS inducer. Our results indicate that exposure to paraquat results in exacerbated cognitive decline in both WT mice and APP/PS1 mice. To determine whether paraquat may regulate expression of the NLRP3 inflammasome to impair cognition, we measured the relative mRNA levels of ASC and Nlrp3, two major components of the NLRP3 inflammasome, in brains from paraquat-exposed animals by qPCR. We found that paraquat-exposed WT mice had higher levels of both ASC and Nlrp3 mRNA than control WT mice. Paraquat-exposed APP/PS1 mice also had increased levels of ASC and Nlrp3 mRNA compared with control APP/PS1 mice. Our results further showed that paraquat treatment increased ASC and Nlrp3 mRNA levels in primary astrocytes. Thus, our data suggest that increased expression of NLRP3 inflammasome may play an important role in mediating cognitive decline induced by mitochondrial ROS.

# Sub article: Non-cell autonomous regulation of body size and metabolism by neuronal mTORC1

**Published:** 2013-12-11

**Authors:** Stacy A. Hussong, Raquel R. Burbank, Linda M. Long, Vanessa Y. Soto, Veronica Galvan

**Affiliations:** 1 Department of Physiology, University of Texas Health Science Center at San Antonio, San Antonio, TX, USA; 2 Barshop Institute for Longevity and Aging Studies, University of Texas Health Science Center at San Antonio, San Antonio, TX, USA; 3 Department of Cellular and Structural Biology, University of Texas Health Science Center at San Antonio, San Antonio, TX, USA

## Abstract

The mechanistic target of rapamycin (mTOR) is a major regulator of cell growth and metabolism. mTOR assembles with Raptor or Rictor to form mTOR complex 1 (mTORC1) and 2 (mTORC2), respectively. Reduced activity of the TOR pathway extends invertebrate lifespan, and, in mice, pharmacologic reduction of mTOR signaling during adulthood, as well as germline reduction of mTOR or S6K1 extending lifespan. In other models of extended longevity, selective reduction of function in the nervous system is sufficient to extend lifespan. The role of mTORC1 signaling from the nervous system in the control of lifespan and metabolism in mammals, however, has not yet been determined. Because neuronal-specific Raptor null mice die perinatally, to explore this question we generated mice expressing a tamoxifen-inducible Cre recombinase in neurons in combination with individual homozygous floxed alleles of genes in the mTORC1 pathway (mTOR^fl/fl^, Raptor^fl/fl^, and S6K1^fl/fl^). Cre recombinase was partially active without tamoxifen stimulation, resulting in the genetic ablation of 20–40% of the target floxed alleles during development. A greater than 20% neuronal knockdown of any of the target genes in the mTORC1 pathway reduced fat content in both sexes. Knockdown of mTOR or Raptor in neurons also significantly reduced body size as well as lower fasting and resting blood glucose levels. These data agree with previously reported data for the germline knockout of S6K1, which also exhibited reduced fat mass, but unlike the germline S6K1^−/ −^ animals, body weight in neuronal-specific S6K1 knockdown mice was unchanged. Only neuronal knockdown of mTOR increased oxygen consumption. Lower glucose and increased lactate in blood were observed in WT and neuronal S6K1 knockdown mice, but not in neuronal mTOR and Raptor knockdown mice challenged with treadmill exercise, indicating altered muscle metabolism as a consequence of neuronal reduction of mTOR or Raptor. Remarkably, S6K1 knockout mice ran farther on the treadmill than WT mice before they reached exhaustion. In contrast, neuronal knockdown of mTOR and Raptor resulted in decreased exercise capacity. Survival to weaning age was reduced but adult mortality was unchanged for Raptor or S6K1 neuronal knockdown mice. Conversely, neuronal mTOR knockdown mice were weaned at the expected Mendelian rates but were short lived, with less than 10% surviving beyond 300 days.

# Sub article: Aldehyde trapping agent, hydralazine, as a potential therapy in Parkinson's disease

**Published:** 2013-12-11

**Authors:** Paul Anthony Martinez, Margaret Chia-Ying Wey, Elizabeth Fernandez, Randy Strong

**Affiliations:** 1 Barshop Institute for Longevity and Aging Studies, University of Texas Health Science Center, San Antonio, TX, USA; 2 Department of Pharmacology, University of Texas Health Science Center, San Antonio, TX, USA; 3 Geriatric Research, Education, and Clinical Center, South Texas Veterans Health Care Network, San Antonio, TX, USA

## Abstract

Parkinson's disease (PD) is a progressive neurodegenerative disorder characterized by the degeneration of nigral dopaminergic neurons leading to motor dysfunction. Several lines of evidence implicate mitochondrial dysfunction, oxidative stress, and protein aggregation in the pathology of PD. Recent evidence, including evidence from our laboratory, suggests that shifts in dopamine and/or aldehyde metabolism lead to aldehyde accumulation, which may contribute to parkinsonism. Aldehyde cytotoxicity may be a result of their long half-life and their ability to cross cell membranes both locally and distant. Aldehyde dehydrogenase (Aldh) is the primary pathway for the detoxification of aldehydes. Mice with mutations for two midbrain isoforms of Aldh, cytosolic Aldh1a1 and mitochondrial Aldh2, exhibit age-dependent neurochemical and behavioral deficits, and respond to levodopa treatment. Aldehyde scavengers such as *N*-benzylhydroxylamine and hydralazine have been shown to provide cytoprotective effects both *in vitro* and *in vivo*. Thus, the detoxification or removal of aldehydes could prove to be effective in PD therapy. The goal of this study is to investigate the protective properties of the FDA-approved anti-hypertensive drug as an aldehyde trapping agent to suppress the parkinsonian phenotype observed in Aldh1a1^−/ −^ X Aldh2^−/ −^ mice. We found that chronic oral administration of hydralazine in drinking water to Aldh1a1^−/ −^ X Aldh2^−/ −^ mice ameliorated both behavioral and neurochemical deficits. In continuance of this investigation, primary cultures isolated from the mesencephalon of Aldh1a1 and/or Aldh2 deficient mice will be subjected to Complex I inhibitors, modeling environmental exposure in PD. Aldehyde load and neuronal death will be assessed in the absence and presence of hydralazine treatment.

# Sub article: Inducing diabetes exacerbates AD-like pathology through an mTOR-mediated mechanism

**Published:** 2013-12-11

**Authors:** Miranda Orr, Angela Salinas, Rochelle Buffenstein, Suri Oddo

**Affiliations:** Department of Physiology, Barshop Institute for Longevity and Aging Studies, University of Texas Health Science Center at San Antonio, San Antonio, TX, USA

## Abstract

Alzheimer's disease (AD) is the most prevalent neurodegenerative disease. The majority of AD cases develop sporadically with unknown etiology. Over the past decade, a strong link between type 2 diabetes (T2D) and AD has been established; however, the mechanisms responsible for this relationship remain unknown. The mammalian target of rapamycin (mTOR) plays a key role in maintaining protein homeostasis, regulating energy metabolism, and diabetic insulin resistance. We hypothesized that mTOR may serve as a mechanistic link between AD and T2D. To explore this potential association, we supplemented the drinking water of 3xTg-AD mice, a transgenic mouse model of AD, with 20% sucrose. A second cohort of mice was given sucrose in conjunction with an mTOR inhibitor, rapamycin. All were compared to age and genotype-matched mice on control diet. We found that the sucrose treatment significantly increased fat mass, impaired glucose tolerance, and altered urine concentration all of which are consistent with physiological changes that occur in T2D. Additionally, sucrose exacerbated AD-like cognitive impairment and Aß and tau brain pathology. Impressively, rapamycin mitigated the sucrose-induced increase in pathology and cognitive impairment. Our results provide compelling *in vivo* evidence that diabetes-associated AD progression occurs through an mTOR-related mechanism. Furthermore, our results indicate that treating diabetic patients with mTOR modulators may decrease their risk of developing AD.

# Sub article: Sustained release of PEDF peptides promotes muscle regeneration

**Published:** 2013-12-11

**Authors:** Yeou-Ping Tsao

**Affiliations:** Department of Ophthalmology, Mackay Memorial Hospital, Taipei, Taiwan

## Abstract

To investigate the effects of pigment epithelium-derived factor (PEDF) peptides on muscle regeneration, a rat myonecrosis model of a single injection of bupivacaine into the soleus muscle was employed. PEDF peptides were delivered by bolus injection of alginate-based sustained release regiment. The muscle fiber proliferation was assayed by the incorporation of BrdU in the proliferating nuclei. The soleus muscle specimens were also stained for satellite cell marker, Pax7, so as to investigate the muscle regeneration activity. Results suggested that the administration enhances the proliferative activities of muscle fibers and/or satellite cells, which in turn may promote the muscle to regenerate. There were higher percentages of muscle fibers containing centrally located nuclei in animals treated with the PEDF peptides than with vehicle. Moreover, on average, diameters of muscle fibers from animals treated with PEDF peptides were larger than those from animals in the blank or bolus control group. These indicated that PEDF peptides stimulate both the proliferation and maturation of muscle stem cells. Gene expression profiling and inhibitor assay on C2C12 cells revealed that the stimulation on muscle progenitor cells by PEDF is mediated by PI3KAKT and mitogen-activated protein kinases (MAPK) p38.

# Sub article: NRIP regulates skeletal muscle contraction and regeneration

**Published:** 2013-12-11

**Authors:** Hsin-Hsiung Chen, Wan-Lun Yan, Szu-Wei Chang, Show-Li Chen

**Affiliations:** Graduate Institute of Microbiology, College of Medicine, National Taiwan University, Taipei, Taiwan

## Abstract

Nuclear receptor interaction protein (NRIP) is a calcium-dependent calmodulin (CaM) binding protein (Ca^+2^/CaM). To investigate the functional role of NRIP in skeletal muscle, we first generated conventional NRIP knock out mice. Here, we demonstrate that NRIP-deficient mice lose muscle strength from the assays of *in vitro* muscle contraction test with neuromuscular blocker to rule out the nerve effect on muscle contraction; plus NRIP-deficient mice show the susceptibility to fatigue during repetitive contraction. Additionally, the exercise performances using rotarod and treadmill tests, NRIP-deficient mice are shown to be weaker than wild-type (WT) mice. We then investigated the mechanisms of NRIP involved in muscle strength and contraction; the results illustrate that NRIP regulates striated muscle function at least via calcineurin phosphatase dephosphorylating NFATc1 and CaMKII phosphorylation to increase slow myosin gene expression and the influx of internal stored Ca^+2^ from sarcoplasmic reticulum by measuring caffeine-induced maximal muscle force. To further investigate the NRIP role in regeneration potential, we then subjected NRIP-deficient and WT mice to unilateral cardiotoxin injection into the tibialis anterior. NRIP-deficient mice show lower centralized nuclei, a lower cell-surface recovery, a decreased area of degenerated tissues, and heterogenous myofiber size compared to weight. In conclusion, NRIP regulates skeletal muscle strength via calcineurin-NFATc1 and CaMKII phosphorylation to regulate muscle excitation/contraction; plus it also has function for regeneration capacity from injured muscles.

# Sub article: A case for indeterminate-growing fish as unique genetic model organisms in aging research, with specific emphasis on sarcopenia

**Published:** 2013-12-11

**Authors:** Jacob Michael Froehlich, Zachary G. Fowler, Nicholas J. Galt, Daniel L. Smith, Peggy R. Biga

**Affiliations:** 1 Department of Biology, University of Alabama at Birmingham, Birmingham, AL, USA; 2 Department of Nutritional Sciences, University of Alabama at Birmingham, Birmingham, AL, USA; 3 Department of Biological Sciences, North Dakota State University, Fargo, ND, USA

## Abstract

As life expectancy rises in developed countries, sarcopenia and dynapenia pose significant threats for the aged, both physically and economically. Current research methods used to study age-related muscle wasting may include limitations, including model organisms that exhibit muscle growth characterized by a pre-defined growth plateau. The lack of model organisms for sarcopenia which exhibit continual growth potential with age has possibly limited advances in understanding this condition for two reasons: (1) the presence of a growth asymptote prevents the discovery of novel genes and/or regulatory pathways involved in the continuation of muscle fiber recruitment throughout adulthood; and (2) the inherent growth plateau may lead to sarcopenia itself, as sustained muscle growth throughout adulthood predicts the presence of myogenic precursor cells resistant to aging. Therefore, we propose a unique muscle growth model for the study of sarcopenia that takes advantage of natural continued muscle fiber recruitment. Evidence from a comparative approach utilizing the subfamily *Danioninae* suggests that the indeterminate growth paradigm of many teleosts arises from adult muscle stem cells with greater proliferative capacity, even in spite of smaller progenitor populations. We hypothesize that paired-box transcription factors, Pax3/7, are involved with this enhanced self-renewal and that prolonged expression of these factors may allow some fish species to escape, or at least forestall, sarcopenia/dynapenia. Future research efforts should focus on the experimental validation of these genes as key factors in indeterminate growth, both in the context of muscle stem cell proliferation and in the prevention of skeletal muscle senescence.

# Sub article: Multicolor fluorescent lineage analysis of satellite cells: a retroviral-based mouse system for studying satellite cell behavior in young and aged skeletal muscle

**Published:** 2013-12-11

**Authors:** Monica N. Hall, Nicole Dalla Betta, Taylor Hotz, Bradley B. Olwin

**Affiliations:** Department of Molecular, Cellular and Developmental Biology, University of Colorado, Boulder, CO, USA

## Abstract

Sarcopenia, the severe loss of skeletal muscle mass and function, is a serious health problem affecting aged individuals. Sarcopenic individuals are weak and frail, which predisposes them to disability and death. The mechanisms responsible for muscle wasting during aging are poorly understood. Muscle maintenance depends primarily on muscle stem cells (satellite cells) that exit quiescence during regeneration and replicate to generate daughter cells that either differentiates to maintain muscle or return to quiescence for self-renewal. Controversy surrounds whether the satellite cell pool is heterogeneous in regards to self-renewal, replication or differentiation. Furthermore, during aging it is unclear whether there is a decline in satellite cell number, function or whether cell fate is altered for a subpopulation of cells. To examine satellite cell heterogeneity *in vivo*, we developed a novel multicolor fluorescent lineage system for studying satellite cell clonal expansion in young and aged mouse skeletal muscle. Our system involves an inducible Pax7-Cre driver to express an avian retroviral receptor specifically in satellite cells, permitting retroviral-mediated gene expression of one of three distinct fluorescent proteins. This highly flexible system has advantages over current multicolor recombination-based systems as the timing of receptor expression and infection can be controlled. This improved multicolor lineage system will permit examination of satellite cell heterogeneity via detection of bias in fluorescence in daughter cells. Characterizing satellite cell behavior *in vivo* will permit us to examine possible defects in cell fates and alterations in subpopulations that may be responsible for the age-related decline in muscle function.

# Sub article: DNA synthesis in cardiac tissue: growth and somatic maintenance in long-lived models

**Published:** 2013-12-11

**Authors:** Karyn L. Hamilton, Joshua C. Drake, Danielle J. Reuland, Frederick F. Peelor, Laurie M. Biela, Richard A. Miller, Benjamin F. Miller

**Affiliations:** 1 Translational Research on Aging and Chronic Disease Laboratory, Department of Health and Exercise Science, Colorado State University, Fort Collins, CO, USA; 2 Department of Pathology and Geriatrics Center, University of Michigan, Ann Arbor, MI, USA

## Abstract

Growth of proliferative cells is dependent on regulation of protein synthesis and subsequent cell division. In post-mitotic tissues such as cardiac and skeletal muscle, growth is accomplished by increased protein synthesis and the donation of nuclear DNA by stem cells. Regardless of proliferative capacity, growth and DNA synthesis are associated with increased protein synthesis. Our central hypothesis is that somatic maintenance, which we define as increased protein synthesis in the absence of growth, is associated with slowed aging. To pursue this central hypothesis, we simultaneously measure both protein synthesis and DNA synthesis using deuterium oxide. Using this approach, we have reproducibly measured DNA synthesis in laboratory rodent cardiac tissue. When comparing several long-lived rodent models, we have shown that the rate of new DNA synthesis in the heart is altered and, in some cases, this is an age-dependent or sex-dependent finding. The observed changes in DNA synthesis in cardiac tissue, which is predominantly populated by terminally differentiated, post-mitotic myocytes, suggests that cardiac muscle has either greater proliferative potential than is perhaps appreciated, or that the new DNA synthesized can be accounted for by donation from cardiac progenitor cells. We will present a comparative analysis of DNA synthesis in cardiac tissue from several long-lived models and the current results of ongoing experiments to identify the origin of the new DNA.

# Sub article: Protection of protein structure and function in the longest lived animal

**Published:** 2013-12-11

**Authors:** Stephen Treaster, Iain Ridgway, Asish Chaudhuri, Steven Austad

**Affiliations:** 1 Barshop Institute for Longevity and Aging Studies, University of Texas Health Science Center at San Antonio, San Antonio, TX, USA; 2 Department of Cellular and Structural Biology, University of Texas Health Science Center at San Antonio, San Antonio, TX, USA; 3 Department of Biochemistry, University of Texas Health Science Center at San Antonio, San Antonio, TX, USA; 4 Department of Molecular Medicine, University of Texas Health Science Center at San Antonio, San Antonio, TX, USA; 5 School of Ocean Sciences, Bangor University, Wales, UK

## Abstract

Traditional aging models are short lived, a useful trait for tracking changes over the course of their lifespan. However, it is within the exceptional long-lived models that we can expect to find the mechanisms necessary to resist aging, as evolution has provided them. To this end, we are utilizing a range of marine bivalve mollusk species, with lifespans ranging from under a decade to over 500 years, in a comparative study to investigate the hypothesis that a long life requires superior proteome stability. These ages can be individually determined by counting growth rings in the shell. This experimental system provides a unique opportunity to study closely related organisms with vastly disparate longevities, including the longest lived animal.

We are testing the long-lived bivalve's ability to maintain protein structure and function under various stressors, and identifying the macromolecules responsible for this protection. As protein homeostasis has been implicated in the aging process and age-related diseases, these macromolecules could have dramatic medical value. Preservation of protein function is measured by representative enzyme activity, such as GAPDH, when stressed *in vitro*. Stressors include both oxidative and unfolding agents. We demonstrate a remarkable persistence in enzyme activity in the long-lived bivalves despite egregious chemical insults. Results also indicate that C57Bl6 exhibit very poor protein stability in comparison to the bivalves. The influence of each species’ metabolite fraction on this stability is also investigated, and an attempt to rescue stability in the short-lived bivalves is attempted. Ultimately, stabilizing compounds will be identified via mass spectrometry. Demonstrating the protection of these proteins and identifying the macromolecules facilitating enhanced proteostasis in the longest lived animal species could be dramatically important in relation to various age-related protein diseases.

# Sub article: Caspase-2 is involved in osteoclast differentiation and apoptosis

**Published:** 2013-12-11

**Authors:** Danielle A. Victor, Jean X. Jiang

**Affiliations:** 1 Department of Cellular and Structural Biology, University of Texas Health Science Center at San Antonio, San Antonio, TX, USA; 2 Department of Biochemistry, University of Texas Health Science Center at San Antonio, San Antonio, TX, USA

## Abstract

Caspase-2 is a protein that is well known for its involvement in cellular apoptosis. However, its function in osteoclasts, cells that resorb bone, has not yet been described. In this work, the role of caspase-2 in osteoclast differentiation and apoptosis is evaluated. During the differentiation process from macrophage precursors, caspase-2 levels decrease so that protein expression is low in the mature osteoclast. Interestingly, when caspase-2 is knocked down in the RAW 264.7 macrophage cell line, osteoclast formation is increased as shown by higher levels of the osteoclast marker Cathepsin K. Furthermore, in cells derived from global *Casp2* knockout mice, osteoclast differentiation is enhanced as demonstrated by increased osteoclast numbers and TRAP activity. With regard to osteoclast apoptosis, cells exposed to oxidative stressors such as H_2_O_2_ and rotenone show increased cleaved caspase-2 protein expression indicating early apoptosis mediated by caspase-2. Furthermore, additional data show that osteoclasts lacking caspase-2 and exposed to oxidative stressors are more resistant to apoptosis compared to wild-type cells. Together, these data suggest that caspase-2 may play a dual role in the osteoclast whereby it modulates osteoclast differentiation and may help regulate osteoclast apoptosis.

# Sub article: Dual recombinase-mediated cassette exchange as an efficient tool to genetically modify mammalian cells

**Published:** 2013-12-11

**Authors:** Feng Li, Riddhi Shah, Eugenia Voziyanova, Yue Li, Yuri Voziyanov

**Affiliations:** School of Biosciences, Louisiana Tech University, Ruston, LA, USA

## Abstract

Genetic manipulations allow controlled interventions into aging progression by changing the genetic composition of model organisms. Such manipulations can include the addition of the ‘anti-aging’ genes, the inactivation of the ‘pro-aging’ genes, or the replacement of the ‘pro-aging’ allelic variants of certain genes with their ‘anti-aging’ counterparts. The genome engineering approaches that can be used to add or replace genes are based on site-specific nucleases and DNA recombinases. Our results indicate that the latter approaches, which currently utilize primarily Flp/*FRT* and Cre/*loxP* systems, can accomplish the task both efficiently and precisely if the fine-tuned dual recombinase-mediated cassette exchange is used as a gene delivery/replacement tool. Under optimal conditions, the efficiency of dual RMCE catalyzed by the Flp–Cre pair can reach about 50% of the transfected cells. Dual RMCE depends on the pre-introduction of the recombination target sites into genome before the replacement reaction can be carried out. This dependence can be lifted if variants of site-specific recombinases are evolved to recognize pre-existing target-like sequences that flank a genome region of interest. Here, we present the results of the engineering of the hybrid Flp-TAL and Cre-TAL recombinases that recognize genomic sequences of interest. These task-specific variants of Flp and Cre recombinases can be paired to be used in the dual RMCE approach to replace the desired genome regions. We show that the engineered hybrid Flp-TAL and Cre-TAL recombinases can be used to mimic the replacement of the mutation that causes sickle-cell anemia in the model setting of CHO cells.

# Sub article: Small-molecule differentiation of marmoset induced pluripotent stem cells

**Published:** 2013-12-11

**Authors:** Steven Farnsworth, Anuja Mishra, Peter Hornsby

**Affiliations:** 1 Department of Physiology, University of Texas Health Science Center at San Antonio, San Antonio, TX, USA; 2 Barshop Institute for Longevity and Aging, University of Texas Health Science Center at San Antonio, San Antonio, TX, USA

## Abstract

Induced pluripotent stem (iPS) cells have the potential to improve health with age. IPS cells may be useful for biological teeth engineering. The critical cells in tooth development are neural crest-derived dental mesenchyme. Here, we utilize pharmacological molecules to assess iPS cells derived from the common marmoset (*Callithrix jacchus*) for the neural tube and neural crest lineage differentiation.

Approach: To assess neural differentiation, we utilized an embryoid body generation assay in combination with a cocktail treatment of active small molecules: DMH1 (BMP inhibitor), SB431542 (activin/nodal/TGF-beta inhibitor), BIO (GSK-3 beta inhibitor), Y-27632 (ROCK/Rho inhibitor) and all-*trans* retinoic acid.

Results: With 6 days of treatment with bFGF withdrawal and GSK-3 beta inhibitor, we found significant increases in the mRNA levels of neural markers *NCAD* (30-fold increase, *P*=0.0001) and *ERBB3* (12-fold increase, *P*=0.0129). We found that bFGF addition had an inhibitory effect on *NCAD*/*ERBB3* induction. When GSK-3 beta inhibitor (BIO) was included, *NCAD* mRNA levels further increased (from a 34- to 102-fold increase over baseline, *P*=0.0001), as was *ERBB3* (34-fold, *P*=0.0035). bFGF withdrawal also increased *FOXA2* levels (endoderm marker), but we report that inclusion of the GSK-3 beta inhibitor (BIO) ablated induction from a 15-fold increase to a 3-fold increase, *P*=0.0318).

Conclusions: Results indicate that FGF inhibits neural differentiation, while GSK-3 beta inhibition promotes neuralization as indicated by *NCAD* and *ERBB3* expression. We report that marmoset iPS cells respond to pharmacological inducers of neural differentiation. These studies will accelerate the development of the marmoset as a model for engineered teeth engraftment.

# Sub article: Reduced systolic cardiac function, but high cardio protection in the long-lived naked mole rat

**Published:** 2013-12-11

**Authors:** Kelly M. Grimes, Anilkumar K. Reddy, Andrew Voorhees, Hai-Chao Han, Merry L. Lindsey, Rochelle Buffenstein

**Affiliations:** 1 Department of Physiology, Sam and Ann Barshop Institute for Aging and Longevity Studies, University of Texas Health Science Center at San Antonio, San Antonio, TX, USA; 2 Division of Cardiovascular Sciences, Baylor College of Medicine, Houston, TX, USA; 3 Department of Mechanical Engineering, University of Texas at San Antonio, San Antonio, TX, USA; 4 Department of Physiology and Biophysics, Jackson Center for Heart Research, University of Mississippi Medical Center, Jackson, MS, USA

## Abstract

The naked mole rat (*Heterocephalus glaber*; NMR) is the longest lived rodent, living >31 years. Unlike aged humans and mice that exhibit large declines in diastolic function, the NMR maintains both systolic and diastolic function for at least 66% of its long lifespan. We have further investigated NMR cardiac function using young (2–4 years) NMRs and have compared them to physiologically age-matched C57BL/6 mice (3–5 months). Surprisingly, we have found that NMRs have low heart rates compared to mice (256±8 vs. 704±11 bpm). NMR heart rates are also about half that predicted for their body size. NMRs display significantly lower cardiac fractional shortening (∼28%) than mice (∼39%). Invasive blood pressure measurements revealed that peak intraventricular pressure and ventricular contractility were much lower in the NMR (*p*<0.001). Histology showed that NMR hearts have reduced myocyte fiber rotation in comparison to the mouse (*p*=0.02). As increased fiber rotation allows for greater torsion, this contributes to decreased NMR heart contractility. Despite these data, predicted systolic wall stress was not different between species, so low systolic function does not necessarily limit wear and tear on the NMR heart. Instead, when compared to mice, NMR hearts have significantly higher levels of cardioprotective factors such as neuregulin-1, Nrf2 and heat shock proteins, which may serve to mitigate structural damage. Overall, the reduced NMR systolic function may be indicative of the species’ low basal metabolic rate, but not necessarily pathology as these rodents maintain cardiovascular function for at least two-thirds of their extraordinary lifespan.

# Sub article: Complex-IV-deficient mice develop increased adiposity and insulin resistance

**Published:** 2013-12-11

**Authors:** Shauna Hill, Daniel Pulliam, Deepa Sathyaseelan, Yuhong Liu, Holly Van Remmen

**Affiliations:** 1 Department of Cellular and Structural Biology, University of Texas Health Science Center at San Antonio, San Antonio, TX, USA; 2 Barshop Institute for Longevity and Aging Studies, University of Texas Health Science Center at San Antonio, San Antonio, TX, USA; 3 GRECC, South Texas Veterans Health Care System, San Antonio, TX, USA

## Abstract

The concept that mitochondrial function declines during aging has been a basic tenet of the biology of aging for many years. However, it has been shown recently, first in invertebrates and later in mice, that lifespan is increased in response to genetic manipulations of some mitochondrial electron transport chain (ETC) complexes. For example, inhibition of complex IV (cytochrome *c* oxidase, COX), an essential transmembrane protein complex in the mitochondrial respiratory electron chain, has been shown to extend lifespan in worms and flies. Similarly, mice lacking the COX assembly protein Surf1 show increased longevity associated with decreased adiposity and enhanced insulin sensitivity, despite 50–70% reduction in COX activity. Here, we asked whether a mouse model of cytochrome *c* oxidase deficiency due to a mutation in the Sco2 gene, a copper chaperone that is required for the activity of COX, would have a similar metabolic phenotype as *Surf1* ^−/ −^ mice. A complete knockout of the Sco2 gene in mice is embryonic lethal, however mice harboring a *Sco2* knockout allele and a mutated *Sco2* knockin allele (*KI/KO*) are viable, and have a 30–60% reduction in COX activity. We found that *Sco2 KI/KO* mice have increased fat mass associated with a reduction in whole white adipose tissue oxygen consumption. The *Sco2 KI/KO* mice have increased hepatosteatosis, elevated serum triglyceride (32%) and cholesterol levels (32%), and changes in circulating adipokine levels compared to wild-type controls. Interestingly, these alterations are associated with the development of insulin resistance in the *Sco2 KI/KO* mice. These findings counter to the metabolic phenotype of *Surf1* ^−*/*−^ mice, illuminating the complex nature of mitochondrial dysfunction on physiology. Results from this study will further enhance our understanding of the role of complex IV in physiological outcomes due to mitochondrial dysfunction.

# Sub article: The regulation of Nrf2 signaling and longevity in long-lived rodents

**Published:** 2013-12-11

**Authors:** Kaitlyn N. Lewis, Rochelle Buffenstein

**Affiliations:** Department of Physiology and the Sam and Ann Barshop Institute for Aging and Longevity Studies, University of Texas Health Science Center at San Antonio, San Antonio, TX, USA

## Abstract

Animal models of extended longevity have a shared characteristic of enhanced cytotoxin resistance. The longest lived rodent, the naked mole rat (∼31 years), is extremely resistant to cancer and a wide array of xenobiotics *in vitro*, and exhibits no age-related physiological or molecular declines during this extraordinarily long lifespan. We hypothesized that this profound broad-based stress resistance was due to enhanced signaling of the nuclear factor-erythroid 2-related factor-2 (Nrf2) cytoprotective pathway. The transcription factor Nrf2 is highly conserved in eukaryotes and ubiquitously expressed in all tissues. Nrf2 levels are regulated by kelch-like ECH-associated protein 1 (Keap1), which targets Nrf2 for ubiquitination and subsequent degradation via the proteasome. After a stressful insult (i.e. toxins, ROS), interactions between Nrf2 and Keap1 are inhibited and Nrf2 is able to translocate into the nucleus, bind to the antioxidant response element (ARE) and thereby activate the transcription of greater than 600 cytoprotective molecules, including those involved in detoxification, glutathione metabolism, molecular chaperones, and proteasome subunits. Commonly studied with regard to cancer, Nrf2 has also been shown to interact with p53 and p21, playing a role in modulation of the cell cycle and cancer progression. We found that Nrf2 signaling activity showed a positive correlation with maximum lifespan in rodents with varying longevity. Interestingly, this was largely due to inverse correlations with several negative regulators of Nrf2, including Keap1, which inhibited degradation of Nrf2. This Nrf2 signaling activity appears to be conserved with aging across 10 different rodent species and the type of regulation may have an impact on positive health span and longevity.

# Sub article: Growth versus somatic maintenance and the synthesis of new DNA in skeletal muscle cells

**Published:** 2013-12-11

**Authors:** Benjamin F. Miller, Joshua C. Drake, Danielle J. Reuland, Frederick F. Peelor, Laurie M. Biela, Richard A. Miller, Karyn L. Hamilton

**Affiliations:** 1 Translational Research on Aging and Chronic Disease Laboratory, Department of Health and Exercise Science, Colorado State University, Fort Collins, CO, USA; 2 Department of Pathology and Geriatrics Center, University of Michigan, Ann Arbor, MI, USA

## Abstract

In proliferative tissues, growth is accomplished with a doubling of cellular machinery by protein synthesis and subsequent cellular replication. In post-mitotic tissues, growth is accomplished by increased protein synthesis followed by DNA donation from stem cells to keep the cyto-nuclear domain constant. In both cases of growth, increased DNA synthesis is associated with increased protein synthesis. Our working hypothesis is that increased somatic maintenance, which we define as increased protein synthesis in the absence of growth, slows down aging. To this end, we have employed methods utilizing deuterium oxide (D_2_O) that simultaneously measure both protein synthesis and DNA synthesis in a variety of tissue types. We have repeatedly demonstrated DNA synthesis in skeletal muscle tissue in human and rodent models. Furthermore, in rodent models of slowed aging (caloric restricted, rapamycin treated, Snell, and crowded litter), we have demonstrated that this rate of new DNA synthesis in skeletal muscle can change based on the model or stage of life. The observable and changeable DNA synthesis in post-mitotic skeletal muscle indicates that skeletal muscle indeed has some replicative potential (as all tissues in mice are telomerase-positive) or that the new DNA comes from stem cells that have replicated and donated DNA to the myocyte. We will present changes in skeletal muscle DNA synthesis in models of slowed aging, and results of studies striving to determine the origin of the new DNA. It is hoped that these studies will provide further insight into how to maximize somatic maintenance for slowed aging.

# Sub article: The paternal age effect: a role for p53 activation in regulating AP endonuclease 1 abundance with increased paternal age

**Published:** 2013-12-11

**Authors:** Jamila R. Momand, Christi A. Walter

**Affiliations:** Department of Cellular and Structural Biology, University of Texas Health Science Center at San Antonio, San Antonio, TX, USA

## Abstract

Over the last few decades, there was a 30% increase in the number of fathers over the age of 35 years. At this age, a father has the potential to transmit twice as many mutations to his child as a 20-year-old father. Paternal mutations continue to double approximately every 16 years, a phenomenon known as the paternal age effect. The driving force of the paternal age effect is mutagenesis. Base excision repair activity is essential for maintaining a low mutant frequency in the male germline. However, spermatogenic cells isolated from old mice display a 50% decrease in base excision repair activity, with a concomitant increase in mutant frequency, as compared to cells prepared from young mice. Reduced base excision repair activity appears to be mediated by reduced AP endonuclease 1 (APE1), a key base excision repair protein. Mice heterozygous for Ape1 show an increased germline mutant frequency as young adults, while APE1 transgenic mice are protected from age-dependent increases in spontaneous mutagenesis. Our objective is to delineate the mechanism/s mediating reduced APE1 abundance in spermatogenic cells with increasing age. In pachytene spermatocytes and round spermatids obtained from old mice, there is a significant 35 and 25% reduction, respectively, in APE1 abundance as compared to young mice. The age-related decrease in APE1 abundance is not accompanied by a reduction in Ape1 transcript abundance, thereby suggesting that post-transcriptional or post-translational regulation is involved. In somatic cells, p53 plays a role in regulating Ape1 abundance. There is a significant increase in p53 activation in spermatogenic cell populations obtained from old mice. APE1 expression is reduced by 40% in spermatogenic populations obtained from p53 null mice, relative to wild-type mice. Combined, these results indicate a strong relationship between APE1 abundance, germline mutagenesis, and p53 activation contributing to the paternal age effect.

# Sub article: Parallel pathways modulate Mit mutant longevity

**Published:** 2013-12-11

**Authors:** Erin Munkácsy, Maruf Khan, Shane L. Rea

**Affiliations:** Department of Physiology, Barshop Institute for Longevity and Aging Studies, University of Texas Health Science Center at San Antonio, San Antonio, TX, USA

## Abstract

Nearly two decades have passed since the initial discovery that disruption of the mitochondrial electron transport chain can unexpectedly increase lifespan. Much research has been done to find the mechanisms behind this seeming paradox, but while many factors have been shown to be required, none has proven sufficient. The mitochondrial unfolded protein response (UPR^mt^) under the control of ATFS-1 has received much attention for its role in Mit mutant longevity, but it is not the only retrograde response induced by mitochondrial dysfunction. We show that the ATFS-1 pathway itself divaricates into separable pathways, one being the UPR^mt^ and the other being the activation of SKN-1. Moreover, we have found a novel retrograde pathway independent of ATFS-1. This MAPK signaling cascade acts in a parallel and compensatory manner with the ATFS-1 network, such that if one pathway is deactivated, the other is turned up to compensate.

# Sub article: Mild calorie restriction diet improves muscle without affecting bone during aging

**Published:** 2013-12-11

**Authors:** Md Mizanur Rahman, Arunabh Bhattacharya, Michael Walsh, Rasel Mohiuddin, Ryan Hamilton, Yuhong Liu, Marian Sabia, Holly Van Remmen

**Affiliations:** 1 Department of Cellular and Structural Biology, University of Texas Health Science Center at San Antonio, San Antonio, TX, USA; 2 Barshop Institute for Longevity and Aging Studies, University of Texas Health Science Center at San Antonio, San Antonio, TX, USA; 3 South Texas Veterans Health Care System, San Antonio, TX, USA

## Abstract

Aging is associated with progressive loss of muscle mass, quality, and strength. Moderate (40–50%) calorie restriction is known to reduce the rate of muscle loss in animal models; however, too high of a calorie-restrictive diet can induce loss of bone mass. In this study, we tested the hypothesis that mild caloric restriction (CR) would still attenuate age-associated loss of muscle mass without any further deleterious effect on bone mass. To determine the long-term effect of mild CR (20% restriction of calorie without reducing vitamins and minerals relative to baseline intake of *ad libitum* (AL)) on musculoskeletal health during aging, 6-month-old C57BL/6 female mice were fed AIN93 diet AL or CR for 8 or 16 months. Mice were scanned with dual energy x-ray absorptiometry (DXA) at 6 months (baseline), 14 months (middle age), and 22 months (aging) and analyzed for the bone and muscle mass. Higher levels of muscle mass are maintained in both 14 and 22 months of age in 20% CR groups as compared to AL groups as measured by DXA, and wet weight at sacrifice. Better muscle strength is also maintained during aging in 20% CR groups as compared to AL groups as measured by rotarod performance test and endurance stress test. Interestingly, bone mass in different bone regions as measured by DXA was not reduced in 20% CR groups as compared to AL groups. These data indicate that 20% CR without compromising vitamin and minerals could be a natural, cost effective, and safe optimal dietary regimen that would improve muscle mass and strength without any deleterious effect on bone.

# Sub article: Adipose precursor cell mitochondrial function differs among sites of adipose depots

**Published:** 2013-12-11

**Authors:** Daniel Pulliam, Arlan Richardson, Adam Salmon

**Affiliations:** UT Health Science Center at San Antonio, GRECC-South Texas Veterans Health Care System, San Antonio, TX, USA

## Abstract

Adipose tissue has dynamic endocrine and secretory functions that play significant roles in the regulation of metabolism and health span. There is increasing evidence that different adipose depots may have different, and often contradictory, effects on this regulation. For example, visceral white adipose is largely thought to be detrimental to metabolism whereas subcutaneous white adipose has been shown to have several beneficial effects. Furthermore, aging is associated with a loss of subcutaneous adipose tissue, accumulation of fat in intra-abdominal visceral adipose tissue and ectopic accumulation of fat in other tissues such as muscle and liver. Adipocyte precursor cells (pre-adipocytes) play a significant role in the homeostatic regulation of adipose tissue. In this study, we tested whether differences in mitochondrial function among the pre-adipocyte pools of each adipose depot might be a determining factor in depot-specific functional differences. Pre-adipocytes from visceral and subcutaneous adipose depots were isolated and sub-cultured from young C57BL/6J mice. Using the Seahorse bioanalyzer, we found that mitochondrial respiration rate is high and reserve capacity is low in visceral depot-derived pre-adipocytes relative to those from subcutaneous depots. In addition, we found that pre-adipocytes from visceral depots are significantly more sensitive to oxidative stress *in vitro* than those from subcutaneous depots. These data then suggest that even modest increases in oxidative stress may dramatically alter the function of adipose precursor cells to inhibit adipogenesis and stimulate lipolysis in visceral depots, which could be a mechanism for increased adipose dysfunction with age. Future studies will address whether alleviation of oxidative stress can preserve healthy functional adipose tissue with age.

# Sub article: The treatment and long-term impact of repetitive traumatic brain injury in mice

**Published:** 2013-12-11

**Authors:** Naomi L. Sayre, Shane Sprague, Murat Digicaylioglu, James D. Lechleiter

**Affiliations:** 1 Department of Cellular and Structural Biology, UT Health Science Center, San Antonio, TX, USA; 2 Department of Neurosurgery, UT Health Science Center, San Antonio, TX, USA

## Abstract

Every year, millions of people are diagnosed with traumatic brain injury (TBI). Of those, many have repetitive TBI (rTBI). Athletes and soldiers are at particular risk for rTBI, and those with rTBI commonly exhibit psychological symptoms including depression, anxiety, poor impulse control, and suicidal ideation. Some also exhibit signs of neuro-degeneration. Currently, treatments to stem the long-term consequences of TBI have been unsuccessful. Astrocytes play a central role in neuronal support and are excellent targets for therapy after TBI. We previously showed that the purinergic agonist 2-methylthioadenosinediphosphate (2MeSADP) effectively reduces astrocyte edema following TBI in mice. We hypothesize that 2MeSADP can similarly reduce the potential long-term effects of repetitive TBI. Here, we report our preliminary findings of a novel rTBI mouse model. Three-month-old mice were subjected to five closed-skull cortical impacts over 5 days. One year after rTBI, behavior was analyzed using a variety of tests. We found that control rTBI mice exhibited more anxiety-like phenotypes compared to 2MeSADP and sham-treated mice in the open field test and the three chambered social anxiety test. Fewer control TBI mice were able to complete the vertical pole test, suggesting an impairment of motor coordination. Continuing experiments will involve histological analysis of brain tissue, and further elucidation of the behavioral phenotype in a second cohort of mice. To our knowledge, this is the first report of a long-term analysis of rTBI in mice.

# Sub article: Effects of aging on derivation of induced pluripotent stem cells

**Published:** 2013-12-11

**Authors:** Jacob Hemmi, Anuja Mishra, Peter Hornsby

**Affiliations:** 1 Department of Physiology, Barshop Institute, University of Texas Health Science Center at San Antonio, TX, USA; 2 Department of Cellular and Structural Biology, Barshop Institute, University of Texas Health Science Center at San Antonio, TX, USA; 3 Audie L. Murphy Memorial VA Hospital, San Antonio, TX, USA

## Abstract

Autologous cell therapies have been proposed as the cure for a wide variety of human pathologies and injuries including diabetes, myocardial infarction, and spinal cord injuries. One obstacle that is often overlooked is that donor age could be a barrier to the derivation of induced pluripotent stem cells (iPSCs) from somatic cells. This is important, as the group for which cell therapy holds the most benefit would be the middle aged to elderly population. It has also been well documented that, during aging, mitochondrial mutations accumulate (due to ROS damage or low-fidelity replication) and oxidative capacity decreases. What, if any, effect these changes would have on the quality of iPSCs derived has yet to be addressed. It has also been shown that in a mouse model carrying a mutation that increases the accumulation of mtDNA mutations, a premature aging phenotype has been shown. Recently, publications have implicated the balance of mitochondrial oxidation to glycolysis as crucial to reprogramming efficiency and have reported that cells acquire high mitochondrial membrane potential during reprogramming. Conflicting reports that this is maintained and leads to increased oxidative capacity after redifferentiation led us to hypothesize that these age-related changes in mitochondrial metabolism play a controlling role in the efficiency of derivation of iPSCs. The current aim is the creation of a polycistronic, floxed retroviral vector to allow for reprogramming with a single virus, which will then be used to investigate effects of donor age on the quality of iPS cells, derived skin samples of marmosets of different ages.

# Sub article: Simultaneous reversal of age-related declines in muscle health and function with transplantation of preconditioned mesenchymal stem cells

**Published:** 2013-12-11

**Authors:** Heather D. Huntsman, Mike DeLisio, Emily Kolyvas, Jenny Merritt, Tushar Bhattacharya, Justin Rhodes, Marni D. Boppart

**Affiliations:** 1 Beckman Institute for Advanced Science and Technology, Urbana, IL, USA; 2 University of Illinois at Urbana-Champaign, Champaign, IL, USA; 3 Department of Kinesiology and Community Health, University of Illinois at Urbana-Champaign, Champaign, IL, USA

## Abstract

Participation in physical activity can effectively prevent muscle loss and functional decline that occurs with age. Thus, understanding the mechanistic basis for preservation of skeletal muscle health with exercise may provide clues for developing therapeutic tools to combat age-related disabilities. We have demonstrated that mesenchymal stem cells (MSCs) accumulate in the muscle of young mice in response to a single bout of eccentric exercise and indirectly facilitate muscle repair and vessel growth via paracrine factor release. The observation that mechanical strain can potently influence growth factor release from freshly isolated, muscle-derived MSCs (mMSCs) provided the impetus to utilize physiological preconditioning as a method to improve mMSC viability and function in the aged muscle microenvironment. In this study, mMSCs isolated from young mouse muscle were preconditioned (10% multiaxial strain, 5 hr) and immediately transplanted into the gastrocnemius/soleus complex of 24-month-old mice. Separate groups of mice receiving saline or non-strained mMSCs served as controls. Although satellite cell number and fiber size were unchanged in all groups, vessel size and number of NMJs were greater in mice injected with preconditioned mMSCs versus controls one week post-injection (*P* < 0.05), resulting in a trend toward an increase in muscle function. Additionally, *in vitro* experiments suggest that preconditioned mMSCs are able to overcome a stiff, collagen-rich microenvironment that mimics aged skeletal muscle, showing increased survival and proliferation as compared to non-strained mMSCs. Overall, MSC preconditioning prior to transplantation may provide a novel method to prevent or reverse age-related impairments in muscle health and function.

# Sub article: Pluripotent minute embryonic-like stem cells and hematopoietic stem cells in murine bone marrow are high in several long-living and reduced in short-living murine strains and unexpectedly, inversely correlated with plasma growth hormone/insulin-like growth factor-1 level

**Published:** 2013-12-11

**Authors:** Magda Kucia, Andrzej Bartke, Mariusz Z Ratajczak

**Affiliations:** 1 Stem Cell Institute at James Graham Brown Cancer Center, University of Louisville, KY, Louisville, USA; 2 Department of Physiology, Southern Illinois University School of Medicine, Springfield, IL, USA

## Abstract

It is known that an increase in caloric intake leads to an increase in plasma growth hormone (GH) level that subsequently induces secretion of insulin-like growth factor-1 (IGF-1) from the liver, what then leads to accelerated aging (*Nature 2010;464:504*). However, caloric restriction and a resulting decrease in plasma IGF-1 level have the opposite effect and extend lifespan. It is also known that adult tissues contain a population of pluripotent very small embryonic-like stem cells (VSELs) that play, as postulated, an important role in the rejuvenation of long-term hematopoietic stem cells (LT-HSCs) in bone marrow (BM) *Leukemia* 2011; doi:10.1038/leu.2011.73, *Exp. Hematology* 2011;39:225–237). As we observed previously, the number of these cells in murine BM decreases with age and VSELs are kept quiescent in BM and protected from premature depletion by the erasure of the somatic imprint in differentially methylated regions (DMRs) of some paternally imprinted genes involved in insulin/insulin growth factors signaling (IIS) such as, for example, Igf2-H19 and RasGRF1 (*Leukemia* 2009;23:2042).

***Hypothesis:*** To explain and connect these phenomena together, we hypothesized that prolonged insulin/insulin growth factor signaling (IIS) prematurely depletes VSELs from the adult tissues and in BM may negatively impact on a population of HSCs.

***Material and methods:*** The number of VSELs and HSCs in long-living murine strains with inborn low levels of circulating IGF-1 (Laron- and Ames-dwarfs) as well as in short-living mice with high levels of circulating IGF-1 (e.g. transgenic mice that overexpress bovine growth hormone; bGH) was evaluated by FACS. VSELs were isolated and the epigenetic status of genes regulating pluripotency (e.g. Oct 4) as well as imprinted genes regulating IIS was evaluated by employing bisulfate modification of DNA followed by sequencing and by COBRE assay. We also challenged long-living mice with low IGF-1 plasma levels by daily injections of recombinant GH or IGF-1.

***Results:*** We found that the number of VSELs and HSCs residing in BM inversely correlates with plasma GH/IGF-1 level. To support this, mice with low circulating plasma IGF-1 levels (Laron- and Ames-dwarf mice) have higher numbers of VSELs and HSCs in BM that, in contrast to age-matched normal litter mates, are maintained at high levels even into advanced age. The analysis of molecular signature of VSELs in these animals revealed prolonged retention of hypomethylation in the DMRs within the Igf2-H19 and RasGRF1 loci, which attenuates IIS signaling in these cells. The number of VSELs, however, decreased in these animals after prolonged treatment with GH or recombinant IGF-I. Conversely, mice with elevated IGF-I level in plasma due to expression of the GH transgene or normal wild-type mice injected for a sustained period with recombinant GH both exhibit significant decreases in the number of VSELs and HSCs in BM compared to control animals. These decreases were paralleled by epigenetic changes in Igf2-H19 and RasGRF1 loci in which DMRs became hypermethylated over time. These changes in methylation lead to increases in IGF-2 and RasGRF1 expression and may explain why GH transgenic mice have an increase in IIS that leads to a shortening of life span in these animals.

***Conclusions:*** Our data shed new light on the relationships between senescence, GH/IGF-1 level, prolonged IIS, and number of VSELs and LT-HSCs. Accordingly, we propose a new paradigm in which a decrease in IIS (e.g. due to caloric restriction that lowers plasma IGF-1 level) may delay the age-dependent elimination of VSELs from adult tissues. In contrast, chronic IIS (e.g. due to chronic high caloric intake and the resulting elevated GH and IGF-1 levels) prematurely depletes VSELs residing in adult organs, which for example in BM leads to a decrease in the number of LT-HSCs. This study also indicates that GH-based anti-aging therapies need careful re-evaluation of their potentially uncontrolled stimulation of VSELs in BM that may lead to development of hematological malignancies. In support of this, elevated GH and IIS lead to hematological malignancies, while, in contrast, Laron-dwarf mice and Laron-dwarf patients, which have low plasma IGF-1 levels, do not develop leukemia.

# Sub article: Tracking the fate of transplanted bone marrow mesenchymal stem cells in Parkinson's disease: a pilot study

**Published:** 2013-12-11

**Authors:** Satish Kumar, Nishi K. Ramana

**Affiliations:** Department of Human Biology/Physiology, International Medical University, Bukit Jalil, Kuala Lumper, Malaysia

## Abstract

Parkinson's disease is a neurodegenerative condition affecting every sixth person older than 70 years in the United States. Current therapeutic approaches provide succour from symptoms, but not a cure. Stem cells, with their propensity to regenerate the dopaminergic neurons (which are actually depleted in this condition) are offering new hope. Steady improvement in the ‘Off’/‘On’ unified Parkinson's disease rating scale (UPDRS), Hoehn & Yahr scores, Schwab and England scores, absence of tumorigenic growth on MRI as well as the absence of any AE's/SAE's following stereotactic transplantation of bone marrow derived mesenchymal stem cells (BM-MSC's) in the sub-lateral ventricular zone (in our earlier study) have infused tremendous optimism and galvanized us to undertake the present, open-labeled, non-randomized, single-center study to evaluate further on the positive outcomes on a larger scale. The current study plans to track the administered BM-MSCs, employing *in vivo* MRI imaging after labeling them with super paramagnetic iron oxide (SPIO), which might help us in confirming and quantifying the success of the transplantation. Also, it might provide early insights on possible mechanisms of any intercellular crosstalk between transplanted BM-MSCs and resident stem cells in the subventricular zone. Finally, this knowledge will help us modify, in future studies, the route of administration, optimize dosage, determine total duration/frequency of transplantation, thus enhancing the therapeutic efficacy. Three of the subjects have shown an 11% improvement in their UPDRS scores ‘Off’/‘On’. There is a marginal improvement in H&Y, as well as H&S scores, in at least five patients. The analysis report of the imaging studies is awaited.

# Sub article: TSH and IL-7 as novel biomarkers expressed by intestinal epithelial stem cells

**Published:** 2013-12-11

**Authors:** Benxu Cheng, Anxiu Kuang, Virginia L. Scofield

**Affiliations:** 1 Department of Cellular and Structural Biology, UT Health Science Center at San Antonio, San Antonio, TX, USA; 2 Department of Microbiology and Immunology, UT Health Science Center at San Antonio, San Antonio, TX, USA; 3 Regional Academic Health Center, University of Texas Health Science Center at San Antonio, Edinburg, TX, USA; 4 Department of Biology, University of Texas, Pan American, Edinburg, TX, USA

## Abstract

In mammals, the small intestines are lined by single-layered epithelium organized into self-renewing crypt-villus units. Dividing stem cells line the valley-like crypts, and these differentiate upward to become quiescent, functional cell types covering the finger-like villi. All of the crypt epithelial cells produce the thyroid-stimulating hormone (TSH) and the cytokine interleukin-7 (IL-7), while villus epithelial cells do not. In the crypts, TSH- and IL-7-expressing cells are layered into three compartments. This layering is not present in knockout mice that do not have intestinal T-cells, suggesting that T-cells are targets for activation via TSH and IL-7, through their surface receptors for both (TSHR and IL-7R). In healthy mice, TSH and IL-7 production are generally limited to the crypts, as noted above, but long contiguous blocks of dividing cells, expressing TSH/IL-7, are present on the villi in intestines of mice infected with enteric pathogens. These reactive cells may be a reserve stem cell population or may have dedifferentiated for T-cell signaling and barrier defense. Dividing cells in the intestinal crypts are recognized targets of agents that cause neoplastic transformation (e.g. ionizing irradiation). We suggest here that dividing, back-differentiated villus cells on the villi may also generate tumors, especially when activating conditions persist, maintaining large numbers of dividing cells on the villi, e.g. in inflammatory bowel syndromes. Targeted delivery of TSH or IL-7 agonists or antagonists may have therapeutic effects for these conditions, via their effects on intestinal T-cells, and may prevent tumor initiation in the intestinal epithelium.
